# Nanocomposite Platform Based on EDTA Modified Ppy/SWNTs for the Sensing of Pb(II) Ions by Electrochemical Method

**DOI:** 10.3389/fchem.2018.00451

**Published:** 2018-10-01

**Authors:** Megha A. Deshmukh, Gajanan A. Bodkhe, Sumedh Shirsat, Arunas Ramanavicius, Mahendra D. Shirsat

**Affiliations:** ^1^Department of Physics, RUSA-Center for Advanced Sensor Technology, Babasaheb Ambedkar Marathwada University, Aurangabad, India; ^2^Jawaharlal Nehru Engineering College, Aurangabad, India; ^3^Department of Physical Chemistry, Faculty of Chemistry and Geosciences, Vilnius University, Vilnius, Lithuania; ^4^Laboratory of Nanotechnology, State Research Institute Center for Physical Sciences and Technology, Vilnius, Lithuania

**Keywords:** ethylenediaminetetraacetic acid (EDTA), nanocomposite, electrochemical detection, conducting polymer, single walled carbon nanotubes

## Abstract

Heavy metal ions are considered as one of the major water pollutants, revealing health hazards as well as threat to the ecosystem. Therefore, investigation of most versatile materials for the sensitive and selective detection of heavy metal ions is need of the hour. Proposed work emphasizes the synthesis of conducting polymer and carbon nanotube nanocomposite modified with chelating ligand for the detection of heavy metal ions. Carbon nanotubes are having well known features such as tuneable conductivity, low density, good charge transport ability, and current carrying capacity. Conducting polymers are the most reliable materials for sensing applications due to their environmental stability and tuning of conductivity by doping and de-doping. Formation of nanocomposite of these two idealistic materials is advantageous over the individual material, which can help to tackle the individual limitations of these materials and can form versatile materials with ideal chemical and electrical properties. Chelating ligands are the most favorable materials due to their ability of complex formation with metal ions. The present work possesses a sensing platform based on conducting polymer and carbon nanotube nanocomposite, which is stable in various aqueous media and possess good charge transfer ability. Chelating ligands played an important role in the increased selectivity toward metal ions. Moreover, in present investigation Ethylenediaminetetraacetic acid (EDTA) functionalized polypyrrole (Ppy) and single walled carbon nanotubes (SWNTs) nanocomposite was successfully synthesized by electrochemical method on stainless steel electrode (SSE). The electrochemical detection of Pb(II) ions using EDTA-Ppy/SWNTs nanocomposite was done from aqueous media. Cyclic voltammetry technique was utilized for the electrochemical synthesis of Ppy/SWNTs nanocomposite. Ppy/SWNTs nanocomposite was further modified with EDTA using dip coating technique at room temperature. The EDTA-Ppy/SWNTs modified stainless steel electrode (SSE) exhibited good sensitivity and selectivity toward heavy metal ions [Pb(II)]. Detection limit achieved for Pb(II) ions was 0.07 μM.

## Introduction

Electrochemical sensors are considered as one of the most prominent and ideal category of sensor technology (Hrapovic et al., 2004; Nemiroski et al., 2014; Dedelaite et al., 2015; Baoyan et al., 2016; Deshmukh et al., 2017; Gu et al., 2018). Organic composite materials are widely used in electrochemical sensors due to their high catalytic activity, large surface area (Cernat et al., 2012; Mittal et al., 2015; Kaisti et al., 2016), ease of synthesis (Xue et al., 2014; Zhanna and Vladimir, 2014), electron transfer and modification by ample number of biochemical and chemical ligands (Oztekin et al., 2011a, 2012a).

Composite structures of conducting polymers and carbon nanomaterials such as carbon nanotubes (CNTs) are especially preferred organic materials for the development of analytical sensors by providing combinational newly formed properties, high sensitivity toward analytes, environmental stability, thus constituting the platform for analytical applications (Teles and Fonseca, 2008). Functionalization of CNTs by organic conducting polymers can greatly enhance their efficacy in the formation of composite structures by aiding in dispersability and ensuring the efficient interaction between CNTs and organic conducting polymers (Ahir et al., 2008). However, the development of simple, cost effective and less effort consuming chemical procedures is always been the demand of the hour (Daniel et al., 2007). Thus, in view of above fact, an environmental friendly, less effort consuming and easy to adopt nanocomposite preparation procedure of SWNTs with polypyrrole (Ppy) was adapted in the present investigation. Polypyrrole was adopted due to good conductivity, stability, efficient polymerization at neutral pH as well as interesting redox behavior, which is suitable for sensor applications (Ahuja et al., 2007; Haghighi and Tabrizi, 2013; Li et al., 2016).

Nanocomposite formation is combination of two or several processes and expected to further improve the deficient properties of each material and resulting material would be with improved features, this improved properties are always advantageous for the development of electrochemical sensors (Xiao and Li, 2008; Dutta et al., 2014). In this context, till date, several studies have been carried out in the area of Ppy and CNTs nanocomposite formation, for example, a nanocomposite of Ppy with p-phenyl sulfonate-functionalized single-walled carbon nanotubes (SWCNTs-PhSO_3_-). The aniline and an oxidative species functionalized SWCNTs was carried out. The composite was used as a platform for the construction of the glucose biosensor (Raicopol et al., 2013). A chemical oxidative polymerization method for the synthesis of a single-walled carbon nanotubes decorated with polypyrrole/TiO2 nanocomposite (Radha et al., 2015). A cavity electrode to synthesize CNTs modified polypyrrole hybrids (Bozlar et al., 2009). The Ppy/MWCNT nanocomposite *in-situ* chemical oxidative polymerization (Bachhav and Patil, 2015). Composite of CNT and Ppy in presence of dopant dodecyl benzene sulfonate (DBS) and Cl- was prepared respectively by chemical oxidation method. The resulting Ppy/CNT composite doped with DBS- and Cl- shows enhanced electrochemical properties (Cai et al., 2016). Above reported Ppy/SWNTs nanocomposite prepared by chemical or electrochemical processes. The processes are time consuming, particular temperature necessities and required more components for the facile synthesis. Chemical route technique is ideal process for material synthesis due to its advantageous characteristics such as large scale and high yield, but chemical process is always time consuming and it requires excessive reducing agents, resulting in hefty chemical residue, and impure nanocomposite (Guo et al., 2009; Wang and Zhang, 2013).

Carbon nanotubes and conducting polymers are considered as versatile and promising materials for research and technological applications. However, there are numerous techniques, which have been reported till date by various research communities for the synthesis of carbon nanomaterials and conducting polymers nanocomposite which exhibits reflux, heating treatments for long time, synthesis in dark conditions etc. (Choi et al., 2011; Abdiryim et al., 2012; Sharma and Sharma, 2013; He et al., 2014). These conditions limit the potential synthesis of nanocomposite materials at room temperature and are also time consuming. Therefore, a future improvement needs better suitable and adaptable materials as well as facile synthesis of carbon nanomaterials and conducting polymers based nanocomposites. The proposed technique offers the easy and less time consuming method for the synthesis of Polypyrrole/single walled carbon nanotubes (Ppy/SWNTs) nanocomposite. It is the most reliable root of materials synthesis over existing chemical methods without excessive heating; it also offers the possibility of utilizing inexpensive and environmental friendly materials and considered as versatile tool for synthesis on lab scale.

Therefore, in the present investigation, we have explored a facile eco-friendly electrochemical method for the synthesis of Ppy/SWNTs nanocomposite at room temperature. In order to understand the electrochemical properties of synthesized material and its use in analytical sensor, further composite structure was modified with ethylenediaminetetraacetic acid (EDTA) and tested for the detection of metal ions [Pb(II)].

Contamination of natural resources due to the metal species is one of the most crucial issues. There are many facts responsible for the pollution of heavy metal ions such as chemical industries, metal plating amenities, mining etc. (Zhang et al., 2010; Oztekin et al., 2011c, 2012b). However, accumulation of heavy metal ions is one of the challenging issues, because they are accumulated within living organisms with imposing adverse effects on the nervous system, immune, reproductive, and digestive systems. Consequently, even trace concentration exposure to various metal ions can lead to long term disorders (Bott, 1995; Darwish and Blake, 2002; Oztekin et al., 2010). Therefore, the accurate and rapid detection of metal species is becoming challenging issue for the research community. Many more methods have been developed for the accurate monitoring of metal species present in drinking water and food chains (Zhang et al., 2010; Oztekin et al., 2011b,d). Despite of staggering number of research papers on the determination of metal ions, investigation of appropriate combination of materials for sensitive and selective detection of metal ions need to focused.

In present research, proposed method enables one step electrochemical synthesis of polypyrrole and single walled carbon nanotubes based nanocomposite (Ppy/SWNTs) and its modification with EDTA as a chelating ligand suitable for the sensitive and selective determination of metal ions particularly Pb(II). The proposed technique has several advantages, (i) EDTA modified Ppy/SWNTs (EDTA-Ppy/SWNTs) nanocomposite is attractive due to its facile, simple and “green” synthesis; (ii) it has a potential to be applied as versatile electrochemical platform for the design of a variety of electrochemical sensors etc.

## Experimental

### Chemicals and reagents

Pyrrole of reagent grade was purchased from Sigma Aldrich (Bangalore, India); Dodecyl benzene sulphonic acid sodium salt (DBSA) was purchased from Kemphasol (Bombay, India) and it was used as a surfactant and organic solvent to form a fine suspension of SWNTs. Lithium perchlorate (LiClO_4_) obtained from Rankem (Bombay, India), SWNTs functionalized with carboxyl groups (-COOH) were purchased from Nanoshel LLC. Ethylenediaminetetraacetic acid (EDTA) was procured from Fisher Scientific, 1-ethyl 3[3(dimethylamino)propyl]-carbodiimide (EDC) was procured from Sigma Aldrich (Bangalore, India). Other chemicals were of reagent grade quality and they were used as received. Stainless steel (SS type 304) purchased from MTI (Korea). An acetate buffer solution (ABS) was prepared by adjusting 0.1 M acetic acid to the desired pH by adding 0.1 M sodium acetate.

### Apparatus

Electrochemical synthesis of Ppy/SWNTs nanocomposite and electrochemical analysis of metal species was performed with a CHI660C computer controlled potentiostat [CH Instruments, Austin, USA). All electrochemical experiments were conducted in a conventional three-electrode cell using the EDTA-Ppy/SWNTs/SS electrode (0.1 mm thick and area 1 × 1 cm^2^)] as a working electrode, Ag/AgCl/KCl (3 M KCl saturated with AgCl) as a reference electrode and Pt plate as a counter electrode. The FTIR analysis was carried out using a Bruker Alpha FT-IR spectrometer (Germany). Raman analysis was carried out using STR 150 Raman spectrometer from Seki technotonics (Germany). X-ray diffraction (XRD) pattern was obtained using Cu source with Bruker D8 Advance diffractometer from Bruker (Germany). The SEM images were obtained by a field-emission scanning electron microscope FESEM Quanta 200 FEG, from FEI Company, (Eindhoven, Netherlands).

### Preparation of Ppy/SWNTs nanocomposite

Ppy/SWNTs nanocomposite was synthesized by an electrochemical method using cyclic voltammetry technique. Briefly, 0.25 M of pyrrole monomer and 0.5 M of LiClO_4_ were added in distilled water (100 ml). 12% wt. of SWNTs has been taken with respect to the concentration of pyrrole monomer in 1 ml of distilled water. DBSA was added as a surfactant in the DI (Deionized water) solution containing dispersed SWNTs to make fine suspension of the SWNTs with the ratio of 10:1 (DBSA:SWNTs) sonicated for 6 h. Resulting suspension of SWNTs was added slowly to the pyrrole+LiClO_4_ solution, and stirred for 20 min at room temperature using magnetic stirrer. The final electrolyte consisted of 0.25 M of pyrrole, 0.5 M of LiClO_4_ and 0.1 mg of SWNTs, and it was utilized in the electrochemical synthesis of Ppy/SWNTs nanocomposite.

Cyclic Voltammetry technique was used for the electrochemical synthesis of Ppy/SWNTs nanocomposite. SS substrate was used as working electrode, Platinum plate as a counter electrode and Ag/AgCl as a reference electrode for the nanocomposite synthesis. The potential was swept between 0.1 to 1.0 V, 5 potential scan cycles at the sweep rate of 0.1 V/s. The composite formation on working electrode was observed by black colored coating with respect to the applied potential and cycles. The deposited black-colored film was washed thoroughly with DI water to remove the excess of monomer from a substrate surface and further dried at room temperature.

### Fabrication of EDTA-Ppy/SWNTs nanocomposite

During the preparation of chelating ligand solution, 0.1 M of EDC (as crosslinking agent) was added to the 0.01 M of EDTA in 100 ml of distilled water and stirred for 20 min. at room temperature. The electrochemically prepared Ppy/SWNTs nanocomposite thin film was incubated in the EDTA solution at room temperature for 5 h. After this time period the EDTA-Ppy/SWNTs modified electrode was rinsed thoroughly with distilled water in order to remove the loosely bound EDTA form the EDTA-Ppy/SWNTs based film.

### Preparation of Pb(II) metal ion solution

For the analysis of metal ions from aqueous media, stock solutions of Pb(II) metal ion was prepared. Pb(NO_3_)_2_ were dissolved in the acetate buffer solutions of pH 4.9. To prepare homogeneous analyte (metal ion) solution it was stirred at room temperature for 30 min. The higher to lower concentration of metal ions was prepared for Pb(II) metal ions in the stock solution.

### Electrochemical detection

DPV was used for the detection of Pb(II) metal ion under optimized conditions. Pb(II) ions were adsorbed on the working electrode by using dip coating technique. The EDTA-Ppy/SWNTs/SSE electrode was incubated for 600 s in the acetate buffer solutions of pH 4.9 containing analyte of Pb(II) ions. The anodic stripping (M^2+^ to M^0^, M = Pb) was performed in the potential range of −0.60 to −0.24 V for Pb(II) ions, with the following optimized parameters: increment of 5 mV, the step amplitude of 50 mV and a pulse period of 0.2 s. The simultaneous and selective detection of Pb(II) ions was performed at the same experimental conditions.

## Results and discussion

### Electrochemical synthesis of Ppy and Ppy/SWNTs nanocomposite

**Figure 1:**
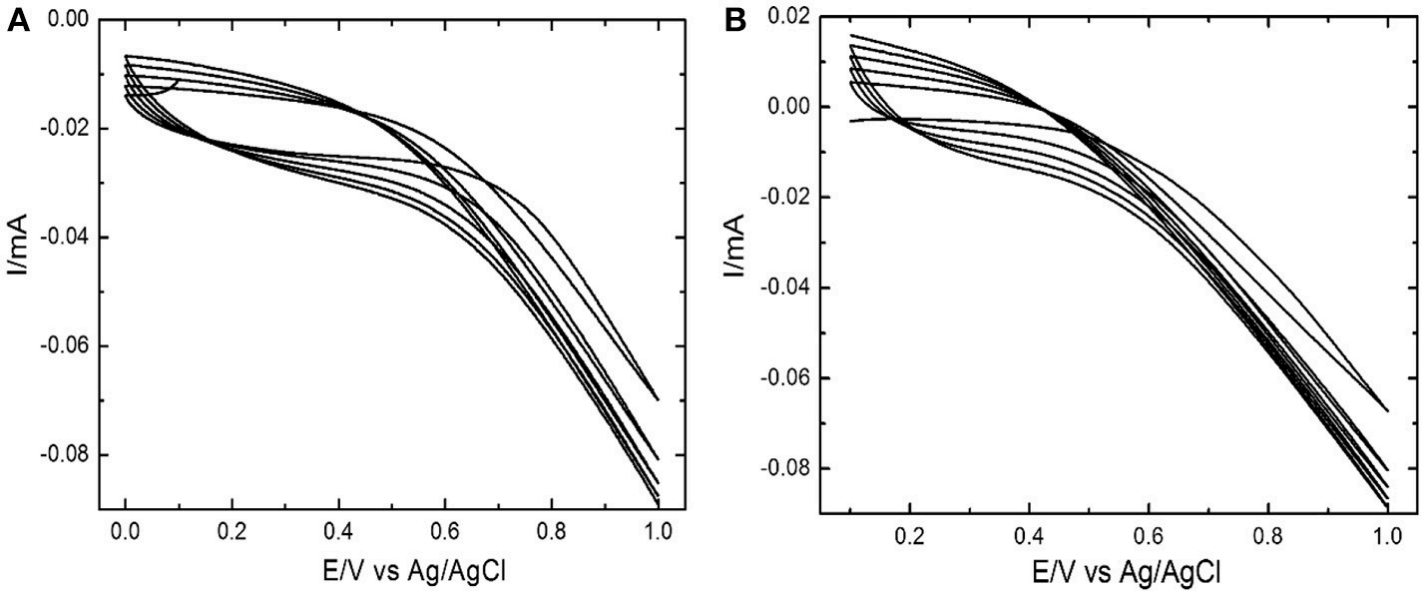
Cyclic voltammogram during electrochemical deposition of unmodified **(A)** Ppy and **(B)** Ppy/SWNTs nanocomposite at a scan rate of 50 mV s^−1^.

Electrochemical polymerization of Ppy and Ppy/SWNTs nanocomposite was performed by potential cycling technique. The cyclic voltammetry (CV) sweeps were recorded for stainless steel electrode during synthesis of unmodified Polypyrrole and Ppy/SWNTs nanocomposite (Figure 1). The cyclic voltammograms of both layers [based on (A) unmodified Ppy and (B) Ppy/SWNTs] showed increase in current density after each successive cycle, which is attributed to electrochemical polymerization of unmodified Ppy (Figure 1A) and Ppy/SWNTs nanocomposite (Figure 1B). Voltammograms of unmodified Ppy and Ppy/SWNTs during synthesis confirms cycle-by-cycle formation of conducting films (Bazzaoui et al., 2005; Santos et al., 2006; Gonzalez and Saidman, 2011; Annibaldi et al., 2012). Indeed, black colored coating was observed at the stainless steel electrode surfaces. The Ppy/SWNTs voltammogram showed increase in current density by each potential cycle compared to voltammograms, which are observed during the formation of unmodified Ppy. Such behavior indicates higher electronic conductivity of Ppy/SWNTs film compared to bare Ppy film. This is due to incorporation of SWNTs. The increase in current density of each material (unmodified Ppy and Ppy/SWNTs) confirms decrease of ohmic resistance for both synthesized films (Mabrouk, 2005; Correia et al., 2007).

### Electrochemical characterization of Ppy, Ppy/SWNTs and EDTA-Ppy/SWNTs nanocomposite

**Figure 2:**
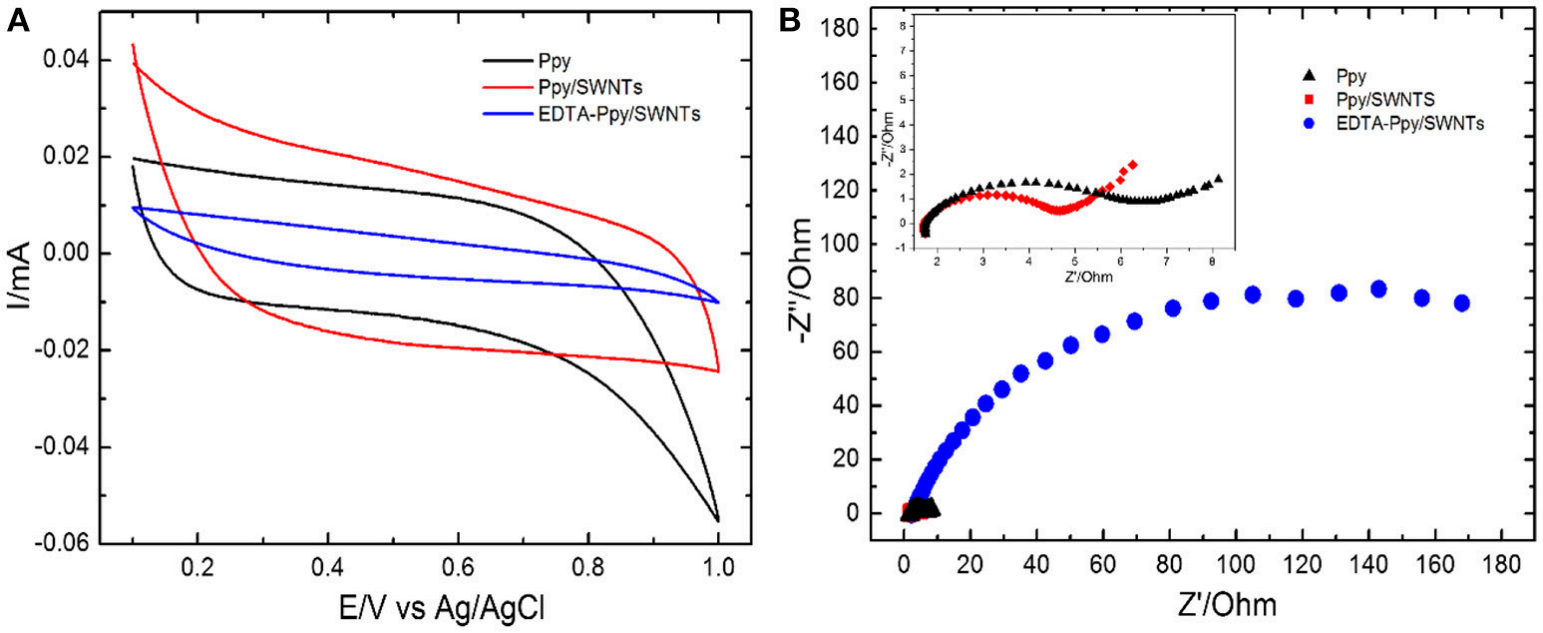
**(A)** Cyclic voltammograms and **(B)** electrochemical impedance spectra (EIS) obtained at unmodified Ppy, Ppy/SWNTs and EDTA-Ppy/SWNTs modified SS in the solution of 0.5 M LiClO_4_.

The comparative electrochemical measurements of Ppy, Ppy/SWNTs and EDTA modified nanocomposite (EDTA-Ppy/SWNTs) nanocomposite are shown in Figure 2. The electrochemical behavior of synthesized materials was studied by cyclic voltammetry in 0.5 M LiClO_4_ (Figure 2A). The unmodified Ppy electrode shows characteristic anodic and cathodic peaks, which are related to the oxidation and reduction of polypyrrole. The onset positions of the unmodified Ppy oxidation peaks are in agreement with the values obtained by cyclic voltammetry, which were registered during synthesis of unmodified Ppy. It was determined that bare Ppy layer has lower oxidation potential compared to that of Ppy/SWNTs nanocomposite layer. EDTA-Ppy/SWNTs nanocomposite structure exhibited lower oxidation potential compared to that of unmodified Ppy and Ppy/SWNTs nanocomposite. Such effect is typically observed due to dominant effect of EDTA to the electrostatic interactions on the surface of modified electrode. The lower current density of EDTA-Ppy/SWNTs nanocomposite can be explained by the complete blocking of the redox reactions and complete coverage of the electrode surface by EDTA.

Electrochemical impedance spectroscopy (EIS), which is a powerful tool for the investigation of modified electrodes, was applied to monitor the unmodified and modified Ppy electrode. EIS based evaluation of unmodified Ppy, Ppy/SWNTs and EDTA—Ppy/SWNTs layers deposited on SS electrodes in 0.5 M LiClO_4_ was performed. The potential amplitude of AC was applied at 0.005 V and the frequency range was set in the range of 1 Hz to 100,000 Hz. Figure 2B shows the Nyquist plots of differently modified electrodes. A single semicircle and a straight line were observed in spectra of unmodified Ppy and Ppy/SWNTs modified SS electrodes. The EDTA-Ppy/SWNTs nanocomposite showed the largest semicircle domain, which illustrates the highest electron-transfer resistance. Comparing EIS spectra of Ppy/SWNTs/SSE with that of Ppy/SSE, a semicircle diameter of EIS spectra of Ppy/SSE represented in Nyquist plot increased due to the absence of SWNTs with Ppy indicating that SWNTs play vital role for the conductivity of Ppy/SWNTs based layers. Furthermore, semicircle in EIS of Ppy/SWNTs/SSE showed substantial decreased in diameter in comparison to EDTA-Ppy/SWNTs nanocomposite. In electrochemical impedance spectroscopy diameter of the semicircle is directly proportional to the resistance (or impedance) to the flow of electrons at the interface (Charge transfer resistance). Larger the diameter, higher is the resistance and lower current flowing in electrochemical cell. Thus, EDTA-Ppy/SWNTs nanocomposite still has good conductivity. These evaluations of EIS spectra are in good agreement with the above CV (Figure 2A).

### Spectroscopic analysis of Ppy, Ppy/SWNTs and EDTA-Ppy/SWNTs nanocomposite layers

#### FTIR analysis

**Figure 3:**
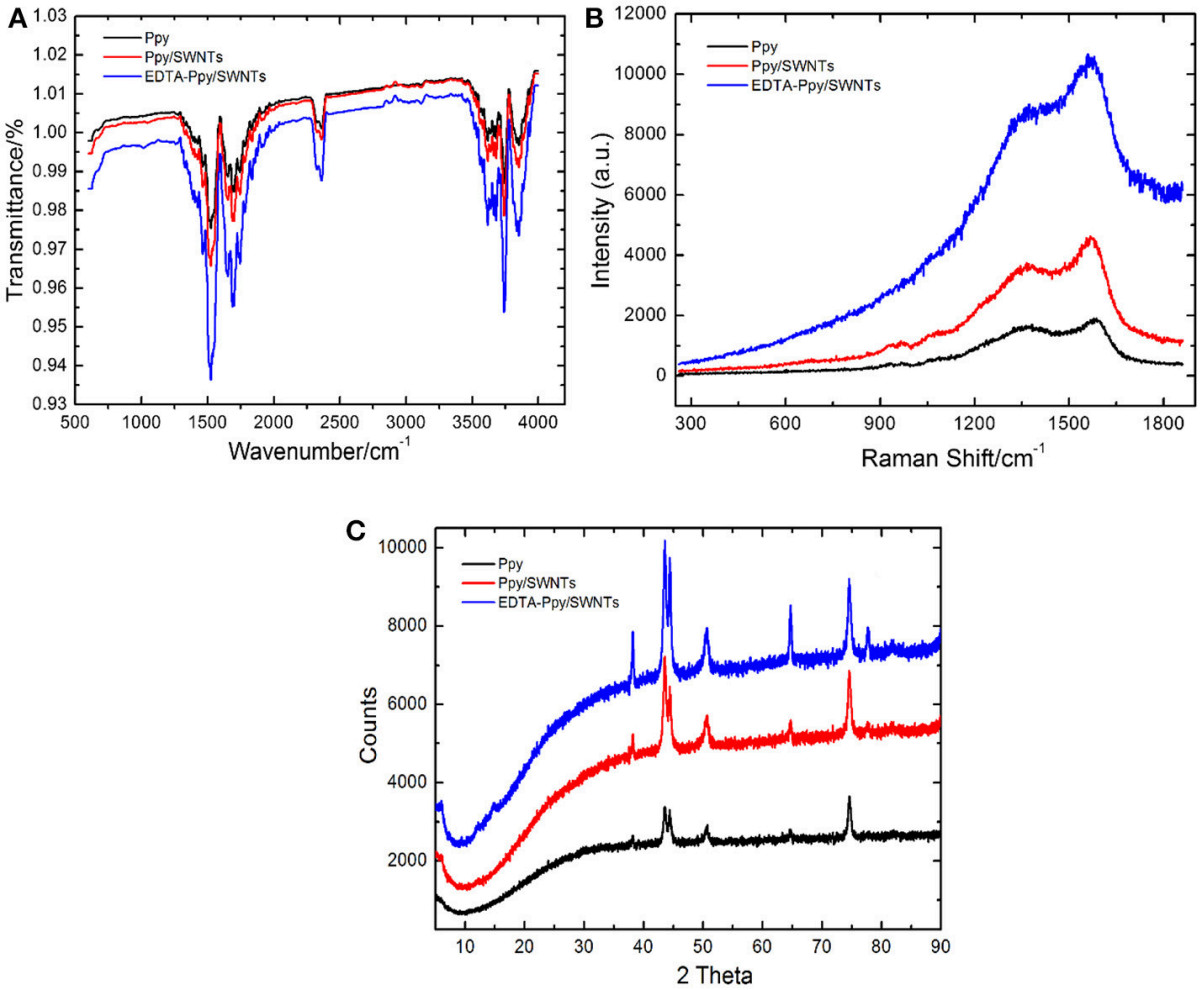
**(A)** FTIR spectra, **(B)** Raman spectra, and **(C)** XRD spectra registered at unmodified Ppy, Ppy/SWNTs, and EDTA-Ppy/SWNTs modified electrodes.

The FTIR spectra registered for unmodified Ppy, Ppy/SWNTs and EDTA-Ppy/SWNTs nanocomposite are shown in Figure 3A. The band at 1291 cm^−1^ is related to the C-N vibrations in-plane, and the bands at 1,175 cm^−1^ and 1,042 cm^−1^ are associated to C-H bending modes while band for C-H out-of-plane deformation vibration was observed at 898 cm^−1^. The FTIR spectra of Ppy/SWNTs nanocomposite corresponds to C = C and C = O stretching at peak intensity of 1,684 cm^−1^ and 1,747 cm^−1^ confirming the oxidized form of SWNTs into carboxylated carbon nanotubes (Liu et al., 2013; Manivel et al., 2014). The characteristic peaks of Ppy at 1,461 cm^−1^ and 1,545 cm^−1^ are hardly seen, which indicates better interaction between aromatic ring of pyrrole and SWNTs. The broad band at 1,745 cm^−1^ for C = O and 3,600 cm^−1^ for O-H were observed in EDTA-Ppy/SWNTs nanocomposite, which confirms the accumulation of EDTA molecules on surface of EDTA-Ppy/SWNTs nanocomposite. It also confirms that EDTA moieties were covalently bounded to surface of Ppy/SWNTs nanocomposite.

#### Raman analysis

The Raman spectra of Ppy, Ppy/SWNTs and EDTA-Ppy/SWNTs nanocomposite are shown in Figure 3B. In spectroscopic measurements Raman spectroscopy has also been utilized for the analysis of surface and interfacial properties of unmodified Ppy, Ppy/SWNTs and EDTA-Ppy/SWNTs nanocomposite. In Ppy, the characteristic Raman bands appeared at about 1583, 1379, 1241, 1070, and 933 cm^−1^ due to the C = C stretching, CC/CN stretching, C–H in-plane bending, and C–H ring deformation, respectively, but their intensity was relatively low compared to the Ppy/SWNTs nanocomposite. The Raman spectrum of Ppy/SWNTs nanocomposite showed two prominent broad characteristics peaks-D band (1368 cm^−1^) and G band (1570 cm^−1^) due to π-π interaction between Ppy and SWNTs. In addition, both these D and G bands shift slightly, when Ppy/SWNTs nanocomposite is modified with EDTA. This effect was observed most probably due to the appearance of carboxyl group of covalently linked EDTA molecules.

#### XRD analysis

The XRD pattern of Ppy, Ppy/SWNTs and EDTA-Ppy/SWNTs are shown in Figure 3C obtained between 2 θ values equal to 5–90°. The XRD of unmodified Ppy shows broad peaks around 2θ 43° indicating the presence of Ppy in the amorphous form due to the amorphous structure of Ppy. The XRD pattern of Ppy/SWNTs shows the broad peak with the range of 2θ 35–45° indicating homogeneous involvement of Ppy in to the structure of nanocomposite. The peak at 43° can be ascribed to the graphite-like structure of SWNTs. The broad peaks within the region of 35–45° indicating the presence of both SWNTs and amorphous polypyrrole which further indicating a homogeneous dispersion of SWCNT in the composite. The new peak observed in XRD pattern of EDTA-Ppy/SWNTs nanocomposite at 2θ 14°.

### Morphological analysis of unmodified Ppy, Ppy/SWNTs, and EDTA-Ppy/SWNTs nanocomposite

**Figure 4:**
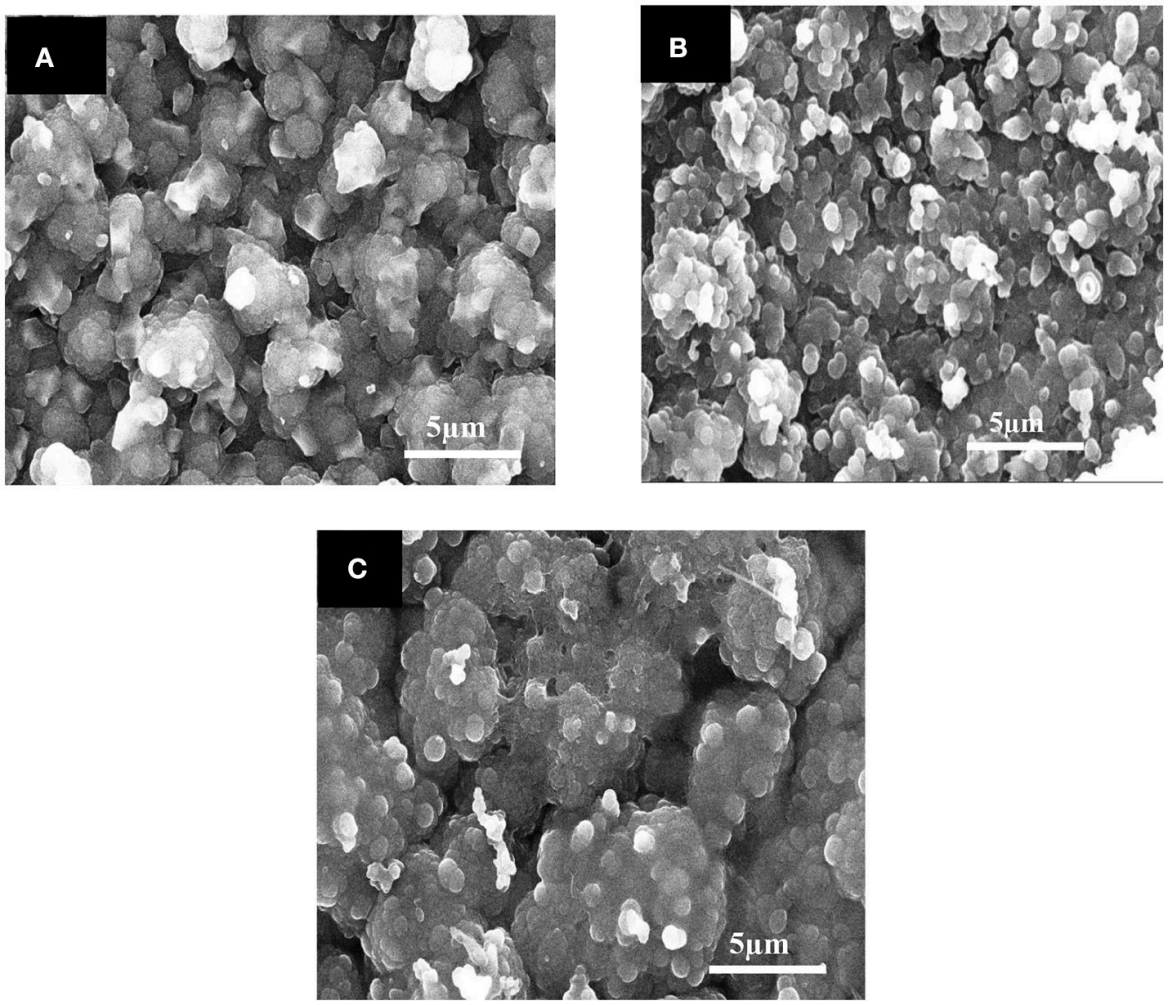
SEM images of **(A)** unmodified Ppy **(B)** Ppy/SWNTs, and **(C)** EDTA-Ppy/SWNTs.

The scanning electron microscopy (SEM) images of unmodified Ppy, Ppy/SWNTs, and EDTA-Ppy/SWNTs nanocomposite are presented in Figure 4. The unmodified Ppy has spherical granular structure (Figure 4A). From the SEM images of Ppy/SWNTs nanocomposite structure it has been clearly seen that Ppy was deposited onto surface of SWNTs retaining its spherical granular structure due to addition of SWNTs content as it is presented in (Figure 4B). It is clearly observed that Ppy is uniformly deposited on the surface of SWNTs, whereas SWNTs can act as backbone of the nanocomposite structure. Figure 4C shows the accumulation of thick layer on the surface of Ppy/SWNTs nanocomposite structure. The SEM of EDTA-Ppy/SWNTs nanocomposite exhibits more granular structure due to accumulation of EDTA molecules on the surface of Ppy/SWNTs structures. Such granular morphology is promising for the accumulation or trapping of analyte due to surface roughness, which is suitable for heavy metal ion sensing.

### Evaluation of sensing parameters

**Figure 5:**
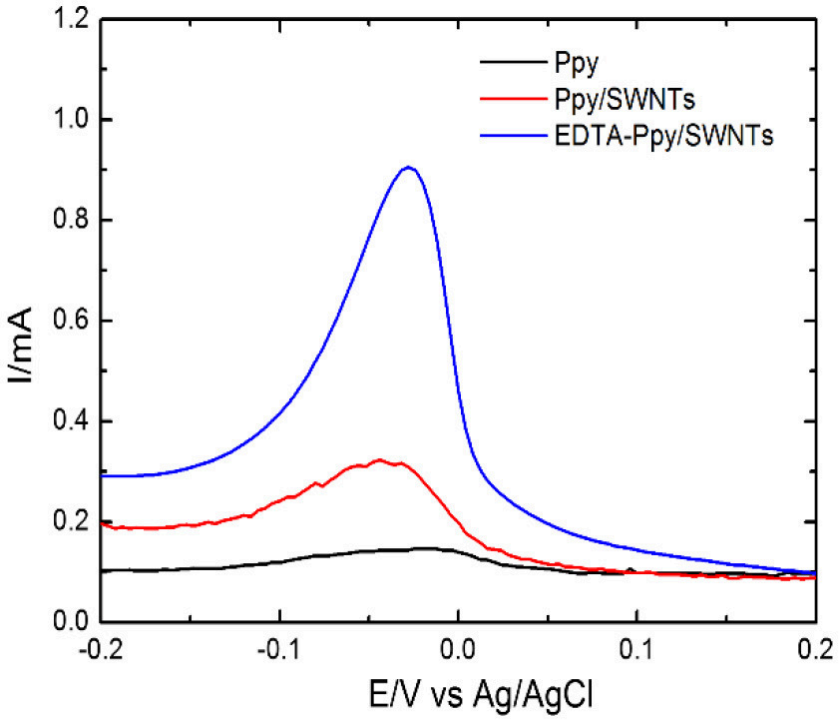
DPV based response of electrodes covered by unmodified Ppy, Ppy/SWNTs and EDTA-Ppy/SWNTs toward 8 × 10^2^ μM Pb(II) ions dissolved in 0.2 M ABS pH 4.9, by the accumulation of metal ions by dip coating technique followed by stripping with DPV technique. The applied DPV parameters *viz*. increment of 5 mV, the step amplitude of 50 mV and a pulse period of 0.2 s in 0.5 M H_2_SO_4_.

In order to achieve best results for the sensitive determination of Pb(II) metal ions with EDTA-Ppy/SWNTs nanocomposite, the sensing parameter *viz* type of sensitive electrode, supporting electrolyte, etc. were optimized in the analyte solution containing 8 × 10^2^ μM of Pb(II) ion. Figure 5 reveals the voltammetric behavior toward Pb(II) with three different electrodes: bare Ppy/SSE, Ppy/SWNTs/SSE and EDTA-Ppy/SWNTs/SSE nanocomposite. The highest sensing peak of Pb(II) was observed in response of EDTA-Ppy/SWNTs/SSE. Thus, EDTA-Ppy/SWNTS nanocomposite based electrode was used in the further experimental analysis.

The sensitive effect of electrodes was studied with unmodified Ppy, Ppy/SWNTs and EDTA-Ppy/SWNTs electrodes (Figure 5) using electrolyte such as acetate buffer, pH 4.9 which was used for the accumulation of Pb(II) ions. In EDTA-Ppy/SWNTs/SSE the EDTA was responsible for increasing selectivity toward Pb(II) ions. However, the EDTA forms complexes with many metal ions. Therefore, in such case synergistic action of all materials applied in sensor design is required in order to increase the selectivity of sensor. In addition, the selectivity of EDTA can be achieved by controlling pH of analyte solution, which is the most proper for the determination of selected metal ions (Rahman et al., 2004).

**Figure 6:**
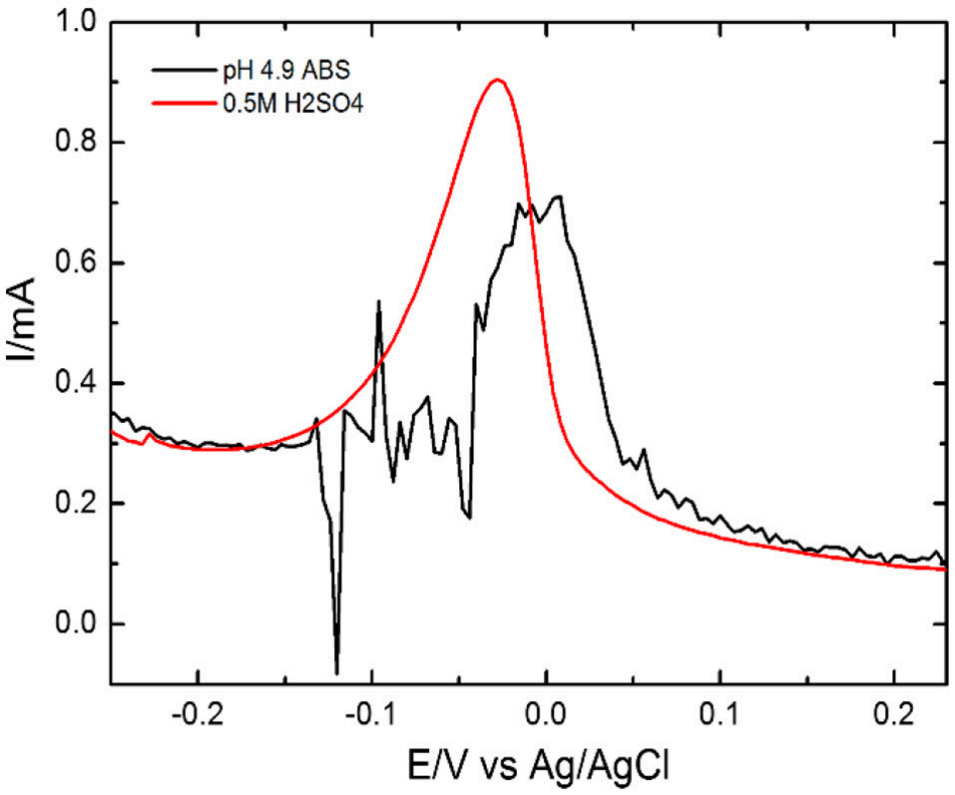
Influence of supporting electrolyte for Pb(II) ions in 0.2 M ABS and 0.5 M H_2_SO_4_ using EDTA-Ppy/SWNTs electrode.

We have selected pH 4.9 for pre-accumulation of Pb(II) ions vs. 0.5M H_2_SO_4_ as an electrolyte for the stripping of Pb(II) metal ions by DPV using EDTA-Ppy/SWNTs electrode. Prior, instead of H_2_SO_4_ electrolyte, we used ABS with pH 4.9 for stripping of Pb(II) ions which was used earlier for the accumulation of Pb(II) metal ion. The electrolyte of acetate buffer of pH 4.9 for Pb(II) metal ion shows the noisy signal without any clear peak as response toward the Pb(II) (Figure 6) ions. It happens due to the acidic media, which is moderately satisfactory environment for the divalent metal ions such as Pb(II) in the presence of EDTA (Reilley and Schmid, 1958). In Figure 6 significant stripping peak current for Pb(II) ion was observed in 0.5 M H_2_SO_4_ based solution_._ Thus, an electrolyte based on 0.5 M H_2_SO_4_ was selected for the further DPV investigations.

### Electrochemical detection of Pb(II) metal ions with EDTA-Ppy/SWNTs-based SS electrode

**Figure 7:**
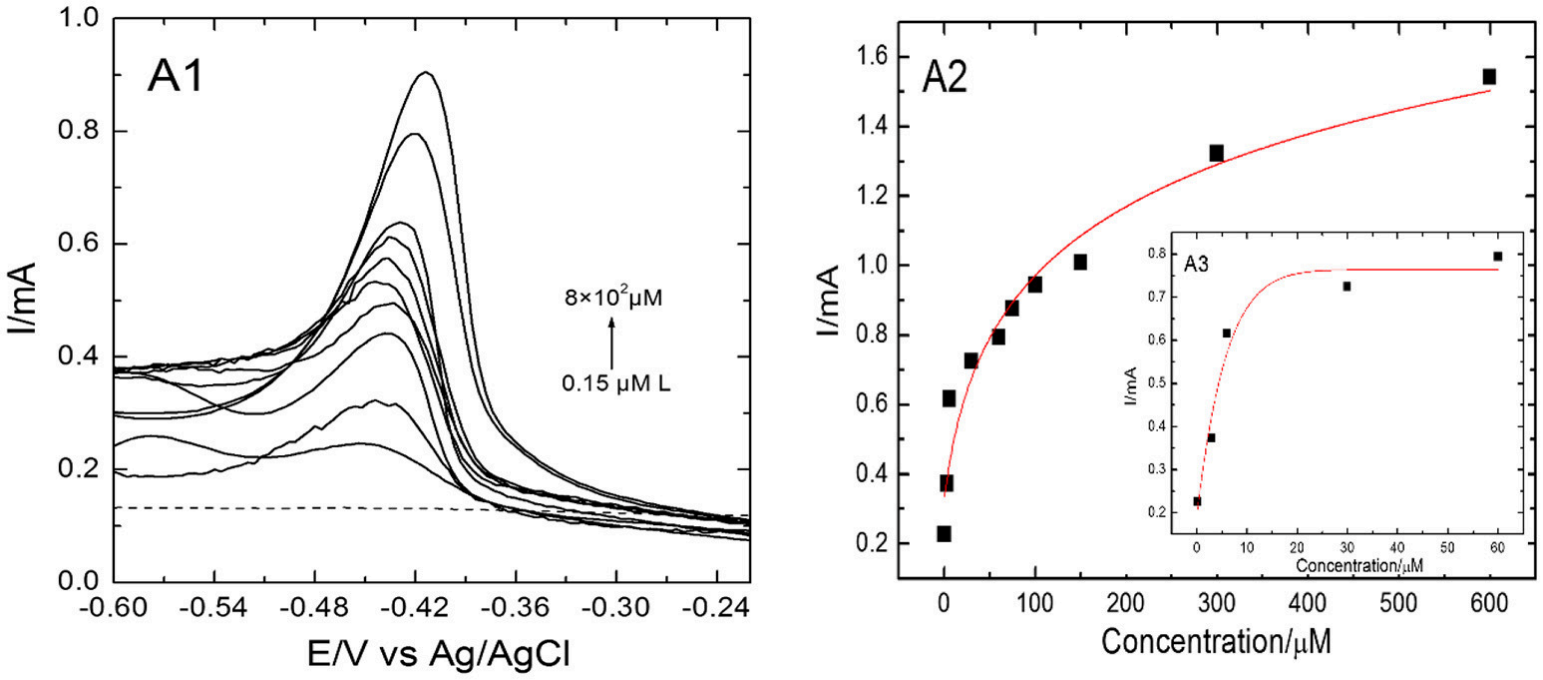
DPV responses and the corresponding hyperbolic calibration plots on EDTA-Ppy/SWNTs modified SS electrode during individual determination of Pb(II) ions within a concentration range of 8 × 10^2^ μM to 0.15 μM for Pb(II) **(A1)**. **(A2, A3)** represent peak currents plotted against Pb(II) concentrations in shorter concentration approximated by hyperbolic equation y = a–b/(1 + c × x)∧(1/d).

Pb(II) metal ions were determined individually and simultaneously by EDTA-Ppy/SWNTs modified SS electrode using DPV in a controlled experimental conditions. Figure 7 shows the DPV response of EDTA-Ppy/SWNTs electrode toward Pb(II) metal ions. As seen from Figure 7, clear DPV response toward Pb(II) over the concentration range from 8 × 10^2^ μM to 0.15 μM in 0.5 M H_2_SO_4_ was observed. The Pb(II) ions were accumulated by dip coating technique for 600 s. Well-defined peaks of Pb(II) ions can be seen in between the −0.60 and −0.24 V for various concentration ranges varied from 8 × 10^2^ μM to 0.15 μM. The detection limit for Pb(II) metal ions was 0.07 μM (according to “3.3 σ method”). The “3.3 σ-based” LOD was calculated from 3.3 × *SD*/*S*, where *SD* is the standard deviation of the measurements and *S* is the slope of the calibration graph. Table 1 represents corresponding parameters of applied hyperbolic equation.

**Table 1:** A1 and B1 represent corresponding parameters of applied hyperbolic equation, supporting electrolyte, 0.5 M H_2_SO_4_.

**Model**	**Hyperbolic Gen**	
Equation	Y = a–b(1 + c*x)∧(1/d)	
Reduced Chi-Sqr.	0.00788	
Adj. R-Square	0.8634	
B1	Value	Standard Error
	a 0.76329	0.0662
	b 0.56927	0.12359
	c − 0.01667	0.40715
	d − 0.09739	2.48416

### Evaluation of interference effects by other metal ions: selectivity study

**Figure 8:**
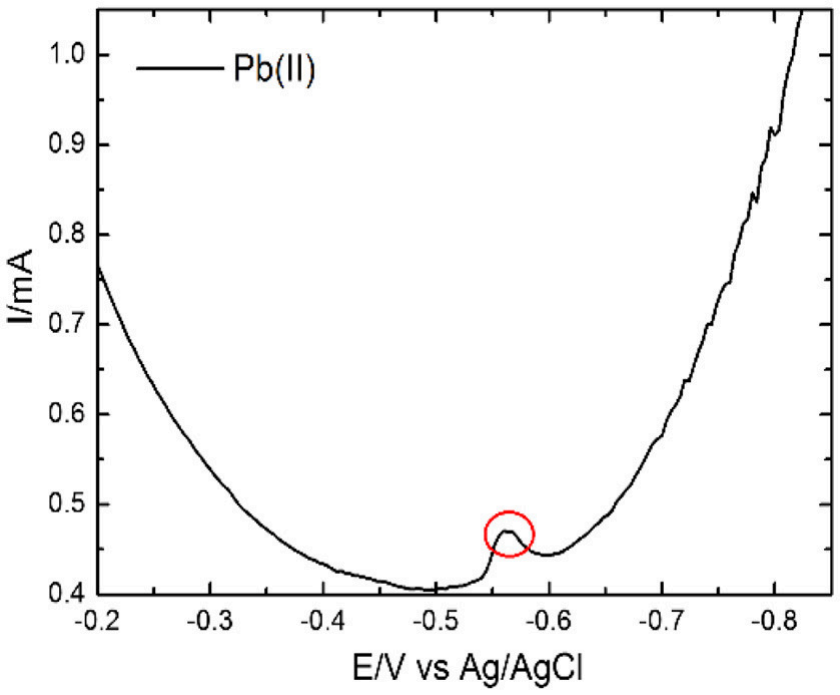
DPV responses on the EDTA-Ppy/SWNTs modified SS electrode for selective detection of Pb(II) ions in presence of the foreign metal ions *viz* Cd(II), Zn(II), Mg(II), Ni(II), and Hg(II) within the concentration range of 8 × 10^2^ μM.

Selective detection of Pb(II) ions was carried out by DPV technique. Prior to this study metal ions pre-concentration was done by dip coating technique for the time period of 600 s at room temperature for the concentration of 8 × 10^2^ μM. For the selective detection of Pb(II) ions, the coexisting interfering metal ions *viz* Cd(II), Zn(II), Mg(II), Ni(II), and Hg(II) were mixed in the acetate buffer solution, pH 4.9, for Pb(II) metal ion. Figure 8 shows selective detection of Pb(II) ions in presence of the mixture of Cd(II), Zn(II), Mg(II), Ni(II), Pb(II), and Hg(II) ions. The selective analysis of Pb(II) shows good results without any interference effect of other metal ions. The observed stripping peak for both metal ions Pb(II) are clear enough to distinguish the exact metal ion.

Comparative study of sensing materials and techniques used for detection of Pb(II) ions reviewed in Table 2.

**Table 2:** Comparative study of the proposed method with other techniques for Pb(II) ion detection.

**Sr. No**.	**Sensing electrode**	**Sensing Technique**	**Detection Limit of Pb(II) ions**	**References**
01	Unfolded fullerene quantum dots	Optical	2.5 μM	Ciotta et al., 2017
02	Nitrogen-doped microporous carbon/Nafion/bismuth-film electrode	Differential pulse anodic stripping voltammetry	0.05 μg L^−1^	Xiao et al., 2014
03	Bismuth modified exfoliated graphite (EG) electrode	Square wave anodic stripping voltammetry	0.83 μg L^−1^	Mafa et al., 2016
04	Size controlled AuNPs/CNFs hybrid	Square wave anodic stripping voltammetry	0.1 μM	Zhang et al., 2016
05	Graphene-polyaniline nanocomposite	Anodic stripping voltammetry	0.1 μg L^−1^	Ruecha et al., 2015
06	Dendritic bismuth film	Square wave stripping voltammetry	5 μg L^−1^	Zhou H. et al., 2016
07	Bi/multiwalled carbon nanotube-emeraldine base polyaniline-Nafion composite modified glassy carbon electrode	Stripping voltammetry	0.08 μg/L	Zhou G. et al., 2016
08	Bismuth/glassy carbon composite	Anodic stripping voltammetry	0.41 μg/L	Hwang et al., 2009
09	Bismuth/electrochemically reduced grapheme/ionic liquid composite modified screen printed electrode.	Square wave anodic stripping voltammetry	0.10 μg L^−1^	Wang et al., 2014
10	Iron oxide/grapheme composite	Differential pulse anodic stripping voltammetry	0.07 μg L^−1^	Lee et al., 2016

## Conclusions

In present work, EDTA modified Ppy/SWNTs nanocomposite was successfully synthesized by electrochemical polymerization method and used as a sensing platform for determination of Pb(II) ions. The spectroscopic, morphological and electrochemical characterization of unmodified Ppy, Ppy/SWNTs, and EDTA-Ppy/SWNTs layers exhibited desired characteristics. EDTA-Ppy/SWNTs exhibits sensitive and selective for detection of Pb(II) ions with lower detection limit of 0.07 μM.

## Author contributions

MS: Provided the intellectual input and designs and approves the protocols to be followed in the study; MD: Carries out the bulk of the experiments, having an important contribution to experimental design, data analysis, interpretation, and writing of the paper; GB: Help in writing the manuscript as well as conducting the experiments; SS: Help in writing the manuscript as well as conducting the experiments; AR: Help in writing the manuscript as well as conducting the experiments.

### Conflict of interest statement

The authors declare that the research was conducted in the absence of any commercial or financial relationships that could be construed as a potential conflict of interest.
